# The anchoring effect and availability bias in healthcare decision-making

**DOI:** 10.3389/frhs.2026.1792537

**Published:** 2026-03-09

**Authors:** Dimitris Zavras

**Affiliations:** Laboratory for Health Technology Assessment, Department of Public Health Policy, School of Public Health, University of West Attica, Athens, Greece

**Keywords:** anchoring effect, availability bias, data, decision-making, probabilistic formalism

## 1 Introduction

Data-driven decision-making (DDDM) refers to the practice of basing decisions on the analysis of data rather than purely on intuition (1). In the context of healthcare, DDDM can improve clinical results, operational efficiency, and financial performance (2).

Data can play an important role in the healthcare sector, especially because of the influence of risk and uncertainty in the decision-making process (3). Improving the quality of healthcare data can impact management and decision-making (4). Typically, data quality concerns accuracy, validity, completeness, comprehensiveness and coverage, reliability, timeliness, linkability, privacy, usability and currency (5). Because information is the end product of data processing (6), we should note that since information quality is dependent on the quality of data (7), in line with their quality, the dimensions of information quality are accuracy, currency, completeness, readability, reliability, usefulness, cost-effectiveness and confidentiality (8).

In addition, poor information quality clearly has a tremendous effect on the efficiency and effectiveness of healthcare organizations at both the operational and strategic levels (9).

However, the critical role of daily decision-making by healthcare managers at all levels is not obvious and well documented (10). This is probably a reason why mistakes in healthcare management have been made. However, making mistakes in all aspects of life is a reality. At the executive level in healthcare, this reality is also true (11), while mistakes can be viewed as making a decision to act or not act without thoroughly assessing known evidence (12). Under this consideration, we may argue that the role of data in healthcare management mistakes is critical.

The anchoring effect is one of the decision-making mistakes attributed to data. It is a common type of error in decision-making and refers to the condition in which decision-makers fixate on initial information as a starting point and then, once set, fail to adequately adjust for subsequent information. First, information carries unwarranted weights relative to the information that is received later. The anchoring effect is linked to time. Availability bias is also linked to time. It occurs when decision-makers tend to remember events that are the most recent and vivid in their memory. Such bias distorts managers' ability to recall events in an objective manner and results in distorted judgments and probability estimates (13).

For example, by using monthly data on economic uncertainty and the number of new confirmed COVID-19 cases and deaths, one can investigate whether governments' decisions regarding policy responses to the pandemic were subject to anchoring and availability biases.

However, since both mistakes have only been conceptually defined, the objective of this study is to develop and present for the first time their probabilistic formalism.

## 2 Probabilistic formalism of the anchoring effect and availability bias

An incorrect decision in a decision-making problem for which a correct solution exists can be characterized as a problem for which needed data are not used. Thus, the probability

$$p_{\mathrm{Incorrect}\;\mathrm{Selection}\;\mathrm{of}\;\mathrm{Alternative}\;\mathrm{Solution}}=\frac{\sum_{t}\sum_{\;j=1}^{\;j=k}(q_{t\;\mathrm{needed}}-q_{t\;\mathrm{not}\;\mathrm{used}|\mathrm{needed}})}{\sum_{t}\sum_{\;j=1}^{\;j=k}q_{t\;\mathrm{needed}}}\in [0,\;1]$$ (1)

corresponds to a condition in which, during the decision-making process, the pieces of multidisciplinary (j=1,2,…,k) information $\;q_{t\;j}s$ that were used, namely,

$$q_{t\;\mathrm{used}}=q_{t\;\mathrm{needed}}-q_{t\;\mathrm{not}\;\mathrm{used}|\mathrm{needed}}$$ (2)

where needed refers to needed information and where not used refers to information that was not used, are not sufficient to correctly solve the problem. Thus, this probability reflects the inability to solve the decision-making problem. In Equation (1), we may assume that $q_{t\;j}s\;$ are defined in terms of time *t*, i.e., the duration of the decision-making process.

The disciplines of information are the same in the numerator and denominator of Equation (1), indicating that an informed decision maker would know what information was needed, i.e., what pieces can solve the problem and then select among them. Under the aforementioned framework, the capacity to solve, namely, the ability of information pieces to solve a decision-making problem, is defined as the probability of such a problem being solved correctly, as shown in Equation (3):

$$p_{\mathrm{Problem}\;\mathrm{Correctly}\;\mathrm{Solved}}=\frac{\sum_{t}\sum_{\;j=1}^{\;j=k}q_{t\;j\;\mathrm{capable}\;\mathrm{to}\;\mathrm{solve}\;\mathrm{the}\;\mathrm{problem}}}{\sum_{t}\sum_{\;j=1}^{\;j=k}q_{t\;j\;\mathrm{used}}}$$ (3)

with $q_{t\;j\;\mathrm{capable}\;\mathrm{to}\;\mathrm{solve}\;\mathrm{the}\;\mathrm{problem}}s\in \;q_{t\;j\;\mathrm{used}}s$ and thus $p_{\mathrm{Problem}\;\mathrm{Correctly}\;\mathrm{Solved}}\;$ takes values in the unit interval. In Equation (3), $q_{t\;j\;\mathrm{used}}$ represents the pieces of information that are used by the decision maker, and $q_{t\;j\;\mathrm{capable}\;\mathrm{to}\;\mathrm{solve}}s$ represents the pieces of information that can correctly solve the problem. In addition, if j=0, the data cannot solve the problem; in other words, information capable of solving the problem does not exist, namely, $p_{\mathrm{Problem}\;\mathrm{Correclty}\;\mathrm{Solved}}$ is not defined (the numerator and denominator are 0). This case corresponds to complete uncertainty. On the other hand, if the decision maker does not consider the total number $(q_{t\;j\;\mathrm{used}}>q_{t\;j\;\mathrm{capable}\;\mathrm{to}\;\mathrm{solve}})\;$ of information pieces, $p_{\mathrm{Problem}\;\mathrm{Correctly}\;\mathrm{Solved}}\;<1$ corresponds to risk, whereas considering the total number of pieces $\left(q_{t\;j\;\mathrm{used}}=q_{t\;j\;\mathrm{capable}\;\mathrm{to}\;\mathrm{solve}}\right)$, $p_{\mathrm{Problem}\;\mathrm{Correctly}\;\mathrm{Solved}}=1$ corresponds to certainty.

Since according to decision-making theory, the outcomes of the decision-making process are functions of uncertainty, risk, or certainty, $p_{\mathrm{Problem}\;\mathrm{Correctly}\;\mathrm{Solved}}\;$ is a catalytic factor in the decision-making process. On the other hand, $p_{\mathrm{Problem}\;\mathrm{Correctly}\;\mathrm{Solved}}$ is linked to decision effectiveness. In this work, effectiveness is defined as the extent to which the targets have been achieved. Thus, we measure effectiveness through probability

$$p_{\mathrm{Effective}\;\mathrm{Through}\;\mathrm{Data}}=\frac{\mathrm{Number}\;\mathrm{of}\;\mathrm{targets}\;\mathrm{that}\;\mathrm{have}\;\mathrm{been}\;\mathrm{achieved}}{\mathrm{Total}\;\mathrm{targets}}\in [0,\;1]$$ (4)

If the problem remains unsolved, $p_{\mathrm{Effective}\;\mathrm{Through}\;\mathrm{Data}}$ is equal to 0.

To solve the decision-making problem, $q_{t\;j}s$ should be equal to $\;q_{t\;j\;\mathrm{needed}}s$.

Due to heterogeneity, when $q_{i}s$ represents different types of information, normalization must be considered. Normalization must discriminate between benefit (higher is better) and cost (lower is better) $q_{i}s$. The max‒min method is the most widely used normalization method. For benefit $q_{i}s$,

$$n_{i}=\frac{q_{i}-q_{\min}}{q_{\max}-q_{\min}}$$ (5)

whereas for cost $q_{i}s$,

$$n_{i}=\frac{q_{\max}-q_{i}}{q_{\max}-q_{\min}}$$ (6)

In addition, weighting methods must also be applied. Although several subjective and objective methods of weighting exist, when there is no information, all weights are considered of equal importance (mean weight method), and they are given by $w_{i}=\frac{1}{m}$ where in our case, *m* represents the number of different types of information. Finally,

$$s_{i}=n_{i}w_{i}$$ (7)

must be calculated, where $s_{i}$ is the weighted normalized score. However, the full representation of normalization and weighting methods is beyond the scope of this paper.

Information used in the anchoring effect is defined as the sum of several pieces of early information, i.e., clinical, administrative, financial, and survey results, during the time period of the decision-making process. Such information can be written as follows:

$$\mathrm{Anchoring}\;\mathrm{Effec}t_{\mathrm{As}\;\mathrm{of}\;\mathrm{time}\;t\;\mathrm{end}}=\sum_{t=t\;\mathrm{initial}}^{t=t\;\mathrm{end}}\sum_{\;j=1}^{\;j=k}s_{t\;\mathrm{early}\;j}$$ (8)

where tend corresponds to the time point of termination of the decision-making process, tinitial corresponds to the time point at which the decision-making process begins and tearly corresponds to data points that are close to the beginning of the decision-making process. Thus, between tend and tinitial, the early information $s_{t\;\mathrm{early}\;j}$ is used, with j∈(0,k] corresponding to the type of data.

Thus, via Equation (8), the probability of the anchoring effect can be written as follows:

$$p_{\mathrm{Anchoring}\;\mathrm{Effect}\;\mathrm{as}\;\mathrm{of}\;\mathrm{time}\;t\;\mathrm{end}\;}=\frac{\sum_{t=t\;\mathrm{initial}}^{t=t\;\mathrm{end}}\sum_{\;j=1}^{\;j=k}s_{t\;\mathrm{early}\;j}}{\sum_{t=t\;\mathrm{initial}}^{t=t\;\mathrm{end}}\sum_{\;j=1}^{\;j=k}s_{t\;j}}$$ (9)

where $s_{t\;j}$ corresponds to information of several types *j* that is available at any time between tinitial and tend. On the basis of Equation (9), lower values of $p_{\mathrm{Anchoring}\;\mathrm{Effect}\;\mathrm{as}\;\mathrm{of}\;\mathrm{time}\;t\;\mathrm{end}\;}$ indicate a greater probability of making a mistake due to anchoring at initial information.

On the other hand, the information used in the availability bias can be written as follows:

$$\mathrm{Availability}\;\mathrm{Bia}s_{\mathrm{As}\;\mathrm{of}\;\mathrm{time}\;t\;\mathrm{end}}=\sum_{t=t\;\mathrm{initial}}^{t=t\;\mathrm{end}}\sum_{\;j=1}^{\;j=k}s_{t\;\mathrm{recent}\;j}$$ (10)

where tend and tinitial are defined as above, while trecent corresponds to a time period where the information is recent and vivid. Thus, $s_{t\;\mathrm{recent}\;j}$ corresponds to information of several types *j* that is recent and vivid.

Using Equation (10), the probability of availability bias can be written as follows:

$$p_{\mathrm{Availability}\;\mathrm{Bias}\;\mathrm{as}\;\mathrm{of}\;\mathrm{time}\;t\;\mathrm{end}}=\frac{\sum_{t=t\;\mathrm{initial}}^{t=t\;\mathrm{end}}\sum_{\;j=1}^{\;j=k}s_{t\;\mathrm{recent}\;j}}{\sum_{t=t\;\mathrm{initial}}^{t=t\;\mathrm{end}}\sum_{\;j=1}^{\;j=k}s_{t\;\mathrm{whenever}\;j}}$$ (11)

where $s_{t\;\mathrm{whenever}\;j}$ reflects information of several types *j* that corresponds to any point in time. Lower values of $p_{\mathrm{Availability}\;\mathrm{Bias}\;\mathrm{as}\;\mathrm{of}\;\mathrm{time}\;t\;\mathrm{end}}$ indicate a greater probability of making a mistake due to availability bias.

As a case study, we can use decision-making, which is based on the bed turnover rate (BTR) and average length of stay (ALOS), since they are both linked to hospitals' efficiency, i.e., hospitals that have a higher BTR and a shorter ALOS are considered more efficient. Both measures are influenced by management and available resources, among other factors. Thus, if solutions are proposed for improving efficiency, the anchoring effect and availability biases must be studied.

Since the BTR is a benefit $q_{t\;j}$, for normalization, Equation (5) can be used, that is,

$$n_{\mathrm{BTR}\;t}=\frac{\mathrm{BT}R_{t}-\mathrm{BT}R_{\min}}{\mathrm{BT}R_{\max}-\mathrm{BT}R_{\min}}$$ (12)

however, since ALOS is a cost $q_{t\;j}$, Equation (6) can be used, that is,

$$n_{\mathrm{ALOS}\;t}=\frac{\mathrm{ALO}S_{\max}-\mathrm{ALO}S_{t}}{\mathrm{ALO}S_{\max}-\mathrm{ALO}S_{\min}}$$ (13)

Assuming equal weights, since the environment is considered uncertain as it was during the COVID-19 pandemic, based on Equation 7,

$$s_{t\;j}=n_{t\;j}w_{t\;j}=n_{t\;j}0.5$$ (14)

Thus, Equations (9) and (11) are written as:

$$p_{\mathrm{Anchoring}\;\mathrm{Effect}\;\mathrm{as}\;\mathrm{of}\;\mathrm{time}\;t\;\mathrm{end}\;}=\frac{\sum_{t=t\;\mathrm{initial}}^{t=t\;\mathrm{end}}(0.5n_{\mathrm{BTR}\;t\;\mathrm{early}}+0.5n_{\mathrm{ALOS}\;t\;\mathrm{early}})}{\sum_{t=t\;\mathrm{initial}}^{t=t\;\mathrm{end}}(0.5n_{\mathrm{BTR}\;t\;}+0.5n_{\mathrm{ALOS}\;t)}}$$ (15)

and

$$p_{\mathrm{Availability}\;\mathrm{Bias}\;\mathrm{as}\;\mathrm{of}\;\mathrm{time}\;t\;\mathrm{end}}=\frac{\sum_{t=t\;\mathrm{initial}}^{t=t\;\mathrm{end}}(0.5n_{\mathrm{BTR}\;t\;\mathrm{recent}}+0.5n_{\mathrm{ALOS}\;t\;\mathrm{recent}})}{\sum_{t=t\;\mathrm{initial}}^{t=t\;\mathrm{end}}(0.5n_{\mathrm{BTR}\;t\;\mathrm{whenever}\;}+0.5n_{\mathrm{ALOS}\;t\;\mathrm{whenever})}}$$ (16)

## 3 Discussion

When sufficient, relevant, and reliable information has been gathered, the decision-making process, which often starts under uncertain conditions, evolves into a more certain process (14). Since information reflects the degree to which uncertainty is reduced (15), uncertainty can be diminished by obtaining relevant information as a result of certain actions, such as not only observing a new fact or performing an experiment but also finding a historical record (16) that is essential for both the anchoring effect and availability bias.

Since strategic decision-making is long-term, information can be quite old (17), meaning that Equations (9) and (11) are valid in terms of timeframe requirements, i.e., age of information (AoI). However, any model constructed from data is subject to the impact of data staleness. Data staleness occurs when the data become outdated to the point that it no longer represents the current real-world scenario; several reasons contribute to data staleness, i.e., changes in business conditions, market dynamics, or customer behaviors, while different scientific fields have varied half-lives (half-life is defined as the time for half of a subject's knowledge to be overturned). Thus, data relevance can be directly impacted by the staleness of the source and types of data reviewed. In this sense, using stale data may lead to incorrect decisions (18–20). Thus, to avoid the anchoring effect as a result of data staleness, decision-makers must recognize the current real-world scenario and its changes. Thus, they must recognize the time point at which the conditions changed. By performing calculations with the help of statistical methods when the user (as a source of truth) should have an updated data element, we can estimate its degree of staleness. On the basis of such estimation, one can distribute resources of an information system in such a way that the repository is partially or entirely as fresh as needed by means of various synchronization techniques (21).

In cases of risk and uncertainty, the probability of making an error is high. In this sense, calculating the probability of making an error provides evidence regarding the correctness of the decision. This gives the decision maker the ability to avoid ineffective decisions. Thus, at an early stage, the decision maker has the opportunity to investigate the outcomes of the decision-making process.

With respect to the healthcare sector, in the era of DDDM, probabilities reflect the opportunity to reconsider the decision-making process to avoid the anchoring effect and availability bias. This, in turn, may result in medical and financial gains for healthcare organizations, improvements in healthcare quality, and, to a greater extent, patient-centered care. The opportunity to reconsider the age of information (AoI) used, as indicated by the probabilities, is a means of eliminating such mistakes.
